# No More Free Drug Samples?

**DOI:** 10.1371/journal.pmed.1000074

**Published:** 2009-05-12

**Authors:** Susan Chimonas, Jerome P. Kassirer

**Affiliations:** 1 Center on Medicine as a Profession, Columbia University, New York, New York, United States of America; 2 Tufts University School of Medicine, Boston, Massachusetts, United States of America

## Abstract

Susan Chimonas and Jerome Kassirer argue that giving out “free” drug samples is not effective in improving drug access for the indigent, does not promote rational drug use, and raises the cost of care.

Everybody likes something free, and free prescription drug samples are no exception. Patients love to receive them, and doctors feel good about handing them out. The practice of providing free drug samples is based on the tacit assumption that “sampling” does much more good than harm. In two separate news releases within the past year by the Pharmaceutical Research and Manufacturers of America (PhRMA), the trade organization that represents the country's largest and leading drug companies, a senior vice president claimed that free samples improve patient care, foster appropriate medication use, and help millions of financially struggling patients. He averred further that samples benefit physicians by exposing them to new treatment options [1],[2]. In this essay, we question the assumption that good trumps harm when prescription drugs are provided free to practicing doctors. We argue that “sampling” is not effective in improving drug access for the indigent, does not promote rational drug use, and raises the cost of care.

## Who Actually Receives the Samples?

Studies show who receives the free drugs. A nationally representative survey in the United States found that fewer than one-third of all sample recipients were low income (defined as less than 200% of the poverty line); by contrast, those in the highest income category were most likely to have received free samples. Moreover, low-income uninsured patients were less likely to receive free samples than those who had continuous health insurance [3]. These findings are supported by an analysis of a large database of individuals who had received at least one prescription drug in a given year. In this study, indigent patients were less likely to receive samples than those classified as non-poor [4].

Often samples never reach their intended audience. Many samples are appropriated by physicians for personal or family use or end up in an “unknown destination” [5],[6]. And in one study, nearly half of pharmaceutical representatives surveyed reported using samples themselves or giving them to their friends and relatives [7]. These studies indicate that samples often reach the wrong people and are frequently misused.

## Quality of Care

Samples can have negative consequences. When low-income patients are given a “starter pack” of samples and a prescription to fill for the remaining period of treatment, they might not be able to afford the cost of the extension, thus leading to discontinuity of treatment. In pharmacies, drugs are labeled, catalogued, stored, and carefully dispensed. In drugstores, pharmacists often identify potentially harmful drug interactions, intercept inadvertent medication errors, and offer a patient-friendly printout of instructions. In doctors' offices, however, detailed patient education regarding sample use rarely occurs, and when it does, it usually lacks information about drug interactions or instructions on how the drug should be taken [8]. Given the lack of oversight by a skilled pharmacist, there is a risk that expiration dates could be overlooked. Moreover, if distribution is inadequately documented in patients' records, some people who receive samples in doctors' offices may not be notified or told to discontinue the medication in the event of a product recall or the emergence of new drug complications.

The samples that drug representatives offer are almost never time-worn and well-tested drugs, nearly never generics, and usually comprise the newest agents on the market. As such, they expose patients to risks not yet identified in clinical trials. The experience with Vioxx is a case in point. By 2002, only three years after Vioxx was introduced, it became the most widely distributed sample [3], and two years later the drug was withdrawn from the market because of an excess risk of myocardial infarctions and strokes [9]. Needless to say, Vioxx was not the only drug given extensively as samples and later found to enhance risk. Samples given to pediatric patients have similarly been associated with notable safety concerns. In 2004, four of the 15 medications most frequently given as samples to children in the US received new or revised “black box” warnings from the US Food and Drug Administration within two years of approval [10]. Finally, patients may not be the only ones at risk from distribution of free samples. Physicians who offer samples to patients and fail to supply appropriate cautions and warnings about the use of these drugs may be subject to liability, along with the company that promoted the drug [11].

## Charity and Education, or Marketing?

It is difficult to escape the conclusion that the prime motivation behind the provision of free samples is marketing. Samples have a major influence on physicians' prescribing habits [12]–[15]. Samples are one of the most effective ways sales representatives get their foot in the door to pitch their companies' products. The technique is effective; the availability of samples is associated with rapid prescription of the new drug [15]. In one study, residents with access to samples were more likely than their counterparts without samples to prescribe heavily advertised products and less likely to suggest an over-the-counter alternative [16]. And a survey based on self-reported physician judgments suggests that the availability of samples might even influence physicians to prescribe drugs that would not otherwise be their top choice [17].

Although the provenance of the casual comments in message boards on Web sites for pharmaceutical sales representatives is uncertain, many of the entries reflect the impressive influence of sampling. Some comments are: “Without samples the available access will be slim to none! The ability to influence doctors will be nil” [18]. Another warned, “If [the companies] are not giving you samples and expecting you to gain access to docs with just a reprint and a detail piece you may be in trouble” [19]. As a physician explained to *The New York Times*, “They are not bringing us samples of things we need…They are bringing us things they want us to know about” [20].

## Health Care Costs

**Figure 1:**
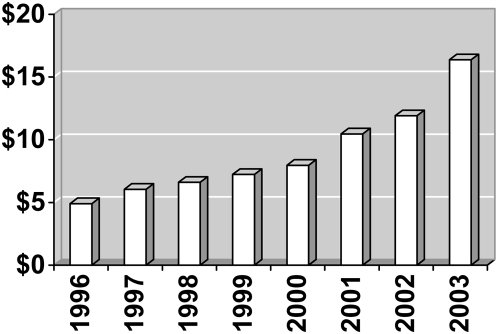
Retail value of US samples, in billions of dollars.

Samples are not effective in lowering patients' costs. Indeed, evidence shows that patients who received free samples had higher out-of-pocket costs than their counterparts who were not given free samples [21]. Samples raise the cost of health care, as companies recoup marketing costs through higher prices and increased sales volume. Samples constitute an enormous promotional outlay of pharmaceutical companies. Between 1996 and 2000, they accounted for slightly more than half of the total promotional dollars spent by industry [22]. Although there is controversy about how best to tally the amount of money the pharmaceutical industry spends on free samples, a recent analysis of 2004 figures sets the retail value of samples at approximately 16 billion US dollars [23]. The retail value of free samples has risen steadily, doubling between 1999 and 2003 [24] (Figure 1). Sample distribution often intensifies during new drug launches, or when a product is withdrawn from the market and competitors scramble to fill the vacuum [25].

**Figure 2:**
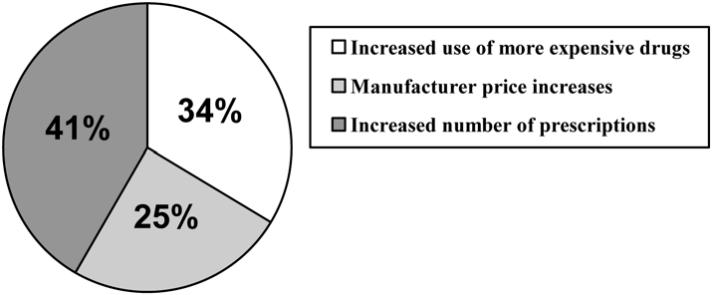
Factors driving growth in drug spending, 1993–2003.

Samples raise health care costs by promoting the use of expensive products. In the US, prescription costs grew 5-fold from 1990 to 2006 [26] and are said to be approaching US$200 billion annually [27]. A substantial fraction of the increase is attributed to a growing reliance on expensive, brand-name medications [28] (Figure 2). One analysis several years ago showed that in a single year, the 50 most heavily marketed drugs accounted for nearly half of the increase in retail spending on prescription drugs (the other 9,850 drugs made up the remaining sum) [29]. These are the very products patients are mostly likely to receive as samples.

## Input from Professional Organizations

A few years ago, a widely acclaimed report on medical errors from the Institute of Medicine took a hard look at free samples. It noted the growing unease in the medical profession about the way samples are distributed, the lack of documentation of medication use, and the by-passing of drug-interaction checks and counseling [30]. Nonetheless, pharmaceutical companies clearly believe that sampling is an effective sales strategy or they would not spend so much of their advertising budget on it, and they continue to distribute samples to individual physicians despite opposition against the practice from a number of groups. The American Society of Health-System Pharmacists, a 35,000-member national professional association of pharmacists in hospitals and health systems, has opposed the use of samples [31]. The Joint Commission, which accredits and certifies more than 15,000 health care organizations and programs in the US, requires that all stages of the medication use process—selection, storage, ordering, dispensing, administration, and monitoring—must be appropriately integrated into a comprehensive medication management system, and this policy applies across the board (K. Powers, personal communication, January 23, 2009). In fact, the Commission reportedly identified lax documentation of drug samples as one of the top ten abuses of hospital-practice standards [32]. The Association of American Medical Colleges and the Association of Faculties of Medicine of Canada recommend distribution of samples only under carefully controlled conditions—namely, by setting up centralized systems at medical centers for accepting and distributing the drugs [33],[34]. These policies are part of a comprehensive approach in North America to reduce the influence of the pharmaceutical industry on the medical profession.

## Alternatives to Samples

“Sampling” is predominately an American problem. Many European countries provide universal health coverage, including prescription drugs. They negotiate with pharmaceutical companies on prices, formally assess the benefits and risks of new drugs, and decide which drugs they will pay for. Under such circumstances, providing new drugs free to doctors has little marketing potential. Discussions abounded at the beginning of 2009 about incorporating many of the health system attributes of European countries into the American health care system, but given the impressive power of lobbyists, many observers doubt that such comprehensive reform will take place. Assuming that the US population will not be covered by a comprehensive prescription drug benefit, alternatives to samples must be available. Many generic and over-the-counter medications are highly effective and have been proven safe by years on the market. They also cost a fraction of the price. RxOutreach.com provides generic drugs to patients at or below 250% of the Federal Poverty Level, at a cost of US$20–US$40 for a 90-day supply [35]. Wal-Mart recently announced that it would offer hundreds of generic drugs for only US$4 per month, and many of its competitors are expected to follow suit [36].

Most pharmaceutical companies offer “patient assistance programs” that provide free or low-cost medications to those who cannot afford them. New resources in the US, including NeedyMeds.com and RxAssist.org, have made information about these patient assistance programs more accessible. Although some patients have found these programs cumbersome, time-consuming, and difficult to navigate, many clinics help patients facilitate their applications.

Many American and Canadian hospitals and medical centers have begun to replace samples with voucher systems. At the University of Michigan, the University of Pennsylvania, the University of Wisconsin, and the Puget Sound Health Alliance, for example, physicians give patients drug vouchers, which are then filled without charge by the hospital pharmacy [37]. This system allows patients to leave the hospital with a trial supply of medication in hand while ensuring adequate documentation and tracking. Yet some US institutions, appreciating the cost implications, have taken a bold step and have barred their physicians from giving free samples or vouchers to patients [38],[39].

## A Call for Change

It is unrealistic to expect pharmaceutical companies to give up one of their most potent marketing techniques voluntarily. Thus, if we are convinced that using free samples is counterproductive in terms of the quality and cost of care, only the medical profession can seek a halt to the practice. The voucher approach is an improvement over our current method of sample distribution, but we favor having our institutions eliminate the use of samples. We call on medical societies, including the American Medical Association, to educate their practitioner members about alternatives to free samples and to re-examine their guidelines on acceptance of samples.

The tradition of physicians dispensing samples has many serious disadvantages and is as anachronistic as bloodletting and high colonic irrigations. As the profession begins to slowly extract itself from the influential grip of industry, it must also deal with the undue influence of free samples.
